# Testosterone-induced benign prostatic hyperplasia rat and dog as facile models to assess drugs targeting lower urinary tract symptoms

**DOI:** 10.1371/journal.pone.0191469

**Published:** 2018-01-19

**Authors:** Jing Li, Yanxin Tian, Shimeng Guo, Haifeng Gu, Qianting Yuan, Xin Xie

**Affiliations:** 1 Chinese Academy of Sciences Key Laboratory of Receptor Research, National Center for Drug Screening, Shanghai Institute of Materia Medica, Chinese Academy of Sciences, Shanghai, China; 2 University of Chinese Academy of Sciences, Beijing, China; IIT Research Institute, UNITED STATES

## Abstract

Benign prostatic hyperplasia (BPH) is an age-related disease, affecting a majority of elderly men worldwide. Medical management of BPH is an alternative to surgical treatment of this disease. Currently, α_1_-adrenergic receptor (α_1_-AR) antagonists are among the first line drugs to treat BPH by reducing the tension of urinary track and thus the obstructive symptoms in voiding. In drug development, old male dogs with spontaneous BPH are considered the golden standard of the animal models. However, old dogs (>6 years) are expensive and not all old dogs develop BPH. So it is necessary to develop more accessible animal models for drug efficacy evaluation. Here we describe the development of testosterone-induced BPH models in both rats and young adult dogs and their applications in the in vivo evaluation of α1-AR antagonist. The BPH rats and dogs induced by chronic testosterone treatment have significantly increased micturition frequency and reduced mean voided volume, very similar to the clinical symptoms of BPH patients. Silodosin, an α1-AR antagonist, significantly reduces the urinary frequency and increases the voided volume in BPH model animals in a dose-dependent manner. The results demonstrate that testosterone-induced BPH rat and dog models might provide a more efficient way to evaluate micturition behavior in anti-BPH drug studies.

## Introduction

Benign prostatic hyperplasia (BPH) is a common disease in middle and old aged men and may significantly affect the quality of life[1, 2]. An estimated 50% of men have histologic evidence of BPH by age 50 and 75% by age 80; in 40–50% of these men, BPH becomes clinically significant[3]. It increases the risk of lower urinary tract symptoms (LUTS), which can be categorized into filling/irritative symptoms (increased urinary urgency and frequency, painful urination, and excessive passage of urine at night) and obstructive symptoms in voiding (poor stream, hesitancy, incomplete voiding, terminal dribbling, and overflow incontinence)[4, 5]. The pathophysiology of BPH, although not fully elucidated, associated with prostate gland overgrowth as a result of androgenic stimulation (static component) and increased adrenergic tone (dynamic component) leading to smooth muscle contraction[6]. Therefore, the anti-BPH drugs can be broadly divided into anti-androgenic drugs (mainly prostate 5α-reductase inhibitors) and anti-adrenergic drugs (mainly α_1_-adrenergic receptors (α_1_-AR) antagonists)[7, 8].

In order to test new anti-BPH drugs, BPH animal models are necessary for *in vivo* efficacy studies. However, spontaneous BPH is rare in species other than man. It has only been described in the dog and chimpanzee[9, 10]. Spontaneous BPH could be observed in male dogs with a prevalence of 16% by age 2, and 50% by age 4–5[11]. Since many features in BPH dogs resemble that in man, old dogs with spontaneous BPH have been used for the study of BPH and to evaluate anti-BPH drugs[12]. Due to the low availability and high cost of old dogs, several *in vivo* experimental BPH models have been developed in other species by hormonal induction, xenografting or transgenic methods[12].

The BPH rat models have been induced by hormones, including androgenic, estrogenic, and progestational hormones [13, 14]. Xenograft models have also been established with cells derived from human BPH tissue culture and primary surgical specimens, which were implanted subcutaneously in immune-deficient rats or mice[15]. Transplants displayed histologically typical BPH acini and stroma[16]. Genetic engineering techniques have also been used in BPH research in recent years. Prolactin transgenic mice develop a significant enlargement in the prostate, which shows similar pathological condition to human BPH[17]. Using transgenic mice, androgen receptor, insulin-like growth factor-1 and a number of other growth hormones have been found to play roles in BPH[18]. These models are typically used to test drugs that can prevent hyperplasia or reduce the size of the prostate.

For α_1_-AR antagonists, their main therapeutic effect is to easy the voiding or obstructive symptoms caused by enlarged prostate. Thus their *in vivo* efficacies were commonly evaluated in rat bladder outlet obstruction (BOO) model established by partial ligature of the proximal urethra[19]. However, apart from the outlet obstruction, the BOO model does not resemble real BPH. It would be interesting to evaluate α_1_-AR antagonists in a model more resemble human BPH, such as a hormonal induced rat BPH model. In addition, we would also like to see whether young adult male dogs could be induced to develop BPH with hormone, and could this model be used to evaluate the effect of α1-AR antagonist.

## Materials and methods

### Animals

Sprague–Dawley rats were purchased from Shanghai Sippr-BK Laboratory Animal Co. Ltd. All rats were raised on a 12 hours dark/light cycle with 3–4 rats in one cage under specific pathogen free condition. Beagle dogs were purchased from the Experimental Animal Institution, Shanghai Jiaotong University Shanghai Sippr-BK Laboratory Animal Co. Ltd. All dogs were raised on a 12 hours dark/light cycle with 1 dog in one cage under clean grade condition.

All experiment procedures for the use and the care of the animals complied with international guidelines for the care and use of laboratory animals and were approved by the Animal Ethics Committee of Shanghai Institute of Materia Medica. All animals were anesthetized prior to experiments with sodium pentobarbital and monitored during the experimental procedure every 10 minutes. After the surgical operation, animals were treated with penicillin for 3 days. No animals died prior to the experimental endpoint. At the end of the experiment, carbon dioxide was used for euthanasia of the rats, and the dogs were put euthanasia by deep pentobarbital anesthesia.

### Chemicals and reagents

Silodosin and testosterone propionate were purchased from Longsheng Chemical (Shanghai, China). Carboxymethyl cellulose sodium was purchased from Guo Yao Regents Co. (Shanghai, China).

### The BPH Rat model

Male Sprague-Dawley rats (200–250 g) were randomly assigned to one of two groups- either castrated or subjected to a sham surgery. After a 7-day recovery, the castrated animals (BPH group, n = 12) were subcutaneously injected with olive oil mixed with testosterone (25 mg/kg per day) for 4 weeks, and sham operated animals (sham group, n = 12) were subcutaneously injected with olive oil.

### The BOO rat model

Female Sprague-Dawley rats (200–220 g) were anesthetized with pentobarbital sodium (50 mg/kg i.p.), and then surgical creation was performed with a previously published method[20]. The abdomen was opened through a midline incision, and the urinary bladder and urethra were exposed. A polyethylene catheter with an outside diameter of 1.09 mm was inserted into the bladder via the urethra. Two silk sutures were placed around the proximal urethra and tied in the presence of the intraluminal indwelling catheter. After these steps, the bladder and prostate were returned to their normal position in the abdomen, and the incision was closed. Sham-operated rats underwent similar procedures without partial urethral ligation. The rats were then allowed to recover, and then housed for 4 weeks with access to food and water ad libitum.

### The BPH dog model

Fourteen adult male Beagle dogs (1 year old, 9–13 kg) were weighted and prostate volume was measured with ultrasonic diagnosis instrument. Micturition behaviors were measured in conscious condition before the dogs were castrated under intravenous pentobarbital anesthesia (pre-BPH group). After a 7-day recovery, the castrated animals were subcutaneously injected with testosterone propionate (10 mg/kg per day) for 8 weeks (BPH group). Every 2 weeks, micturation parameters (urinary frequency, mean voided volume) were measured in the conscious dogs until these parameters suggested the BPH model has been established successfully. After that, the prostate volume was measured again with ultrasonic diagnosis instrument.

### Micturition recording

Micturition behavior was measured after the induction of BPH in both rats and dogs. We divided the animals into 3 groups: sham-operated, vehicle-treated, and silodosin-treated. Silodosin were tested in four doses (0.3, 1, 3, and 10 mg/kg). Two days after a lower-dose testing, animals were used for the next higher-dose testing. BPH rats and BPH dogs were weighed and silodosin was given via oral administration, and 0.5% carboxymethyl cellulose sodium was given as vehicle control. Twenty minutes after compounds administration, animals were given distilled water (30 ml/kg) orally. Immediately after water loading, each animal was placed in a metabolic cage in which the urine was directed into collectors on an electronic balance (YP2002, Yueping Co, Ltd, Shanghai, China). Micturition frequency and mean voided volume were monitored for 2 hours.

### Histopathological examination

Fixed prostate tissue embedded in paraffin wax was cut into 5-μm-thick sections and stained with hematoxylin and eosin. The sections were mounted and cover slipped using mounting solution and then examined under a microscope.

### Data analysis

Data were analyzed with GraphPad Prism software (GraphPad). Means ± SEM were calculated using this software. Student t test was used to evaluate the statistical difference between two groups. One-Way ANOVA followed by Newman-Keuls post hoc test was performed to evaluate the differences among multiple groups.

## Results

### Testosterone propionate induces BPH in rats

**Fig 1:**
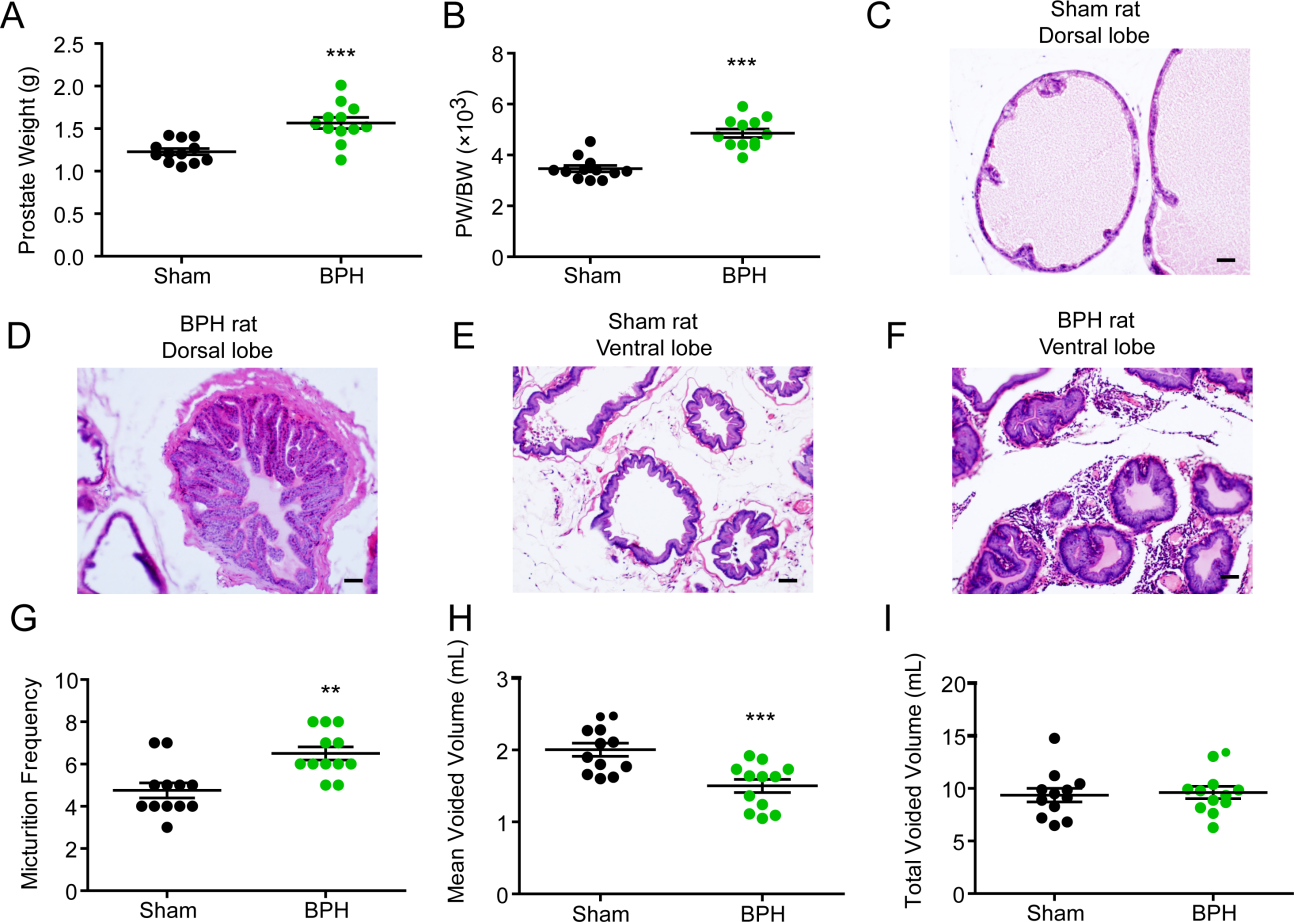
Voiding behavior in testosterone-induced BPH rats. The rat BPH model was generated by testosterone injections for 4 weeks after castration. Sham: sham operated + olive oil injection; BPH: castration + testosterone propionate injection (dissolved in olive oil, 25 mg/kg, once daily). (A) Prostate (containing the ventral, lateral and dorsal lobes) weight and (B) prostate weight to body weight ratios (PW/BW) of sham and BPH rats. H&E stains of dorsal lobe (C and D) and ventral lobe in shame rats and BPH rats (E and F). (G) Micturition frequency, (H) mean voided volume and (I) total voided volume were measured in sham and BPH rats in the first 2 hours after water load (30 ml/kg). **P < 0.01, ***P < 0.001 versus sham control (Student t test). Scale bar, 50μm.

To evaluate the *in vivo* efficacy of α_1_-AR antagonists on the micturition behavior in BPH animals, we first developed hormone-induced BPH model in rats. Male SD rats (200–250 g) were castrated. After recovery, they were given testosterone propionate (25 mg/kg, once daily) for 4 weeks by subcutaneous injection. Compared to those isolated from sham-operated animals, the prostates from the testosterone-treated animals had significantly increased size, weight (Fig 1A) and prostate weight to body weight ratio (Fig 1B). As shown in Fig 1C–1F, the histological feature of the prostate tissue in the BPH group was significantly altered. The prostate epithelial cell layer and stromal cell space in both the dorsal (Fig 1D) and ventral lobes(Fig 1F) of the BPH rats were larger than those of the sham rats. Similar to BPH patients, testosterone-induced BPH model rats have significantly increased micturition frequency (Fig 1G) and significantly reduced mean voided volume (Fig 1H) as compared with the sham-operated rats. No significant difference was observed in the total voided volume between the BPH and sham groups (Fig 1I). Low doses of testosterone propionate (5 or 10 mg/kg, once daily) were also used to induce BPH model, but failed to produce any significant effects on micturition behavior (data not shown).

### Silodosin improves micturition parameters in testosterone-induced BPH rats

**Fig 2:**
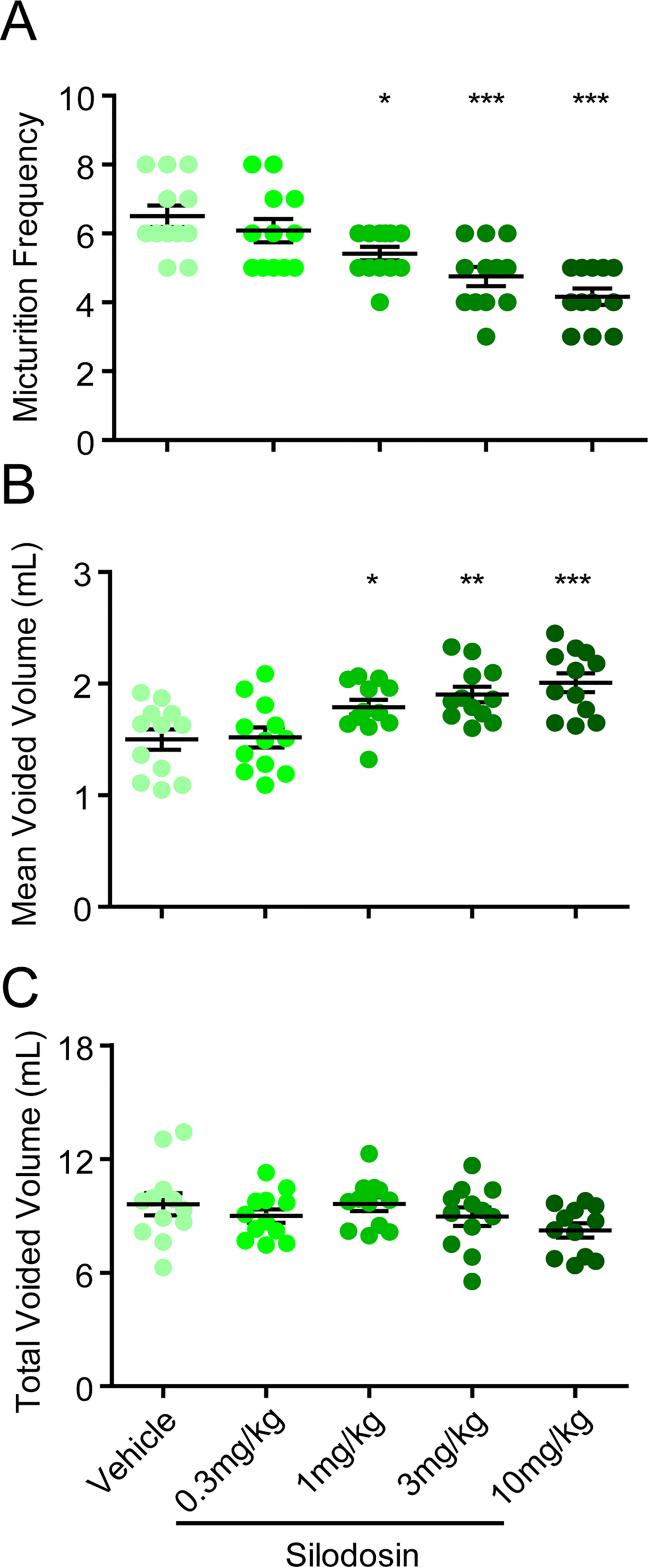
Effects of silodosin on the micturition parameters in testosterone-induced BPH rats. Twenty minutes after oral administration of silodosin, distilled water (30 ml/kg) were given orally, and the micturition parameters of BPH rats were measured for the first 2 hours after water load. (A) Micturition frequency, (B) mean voided volume and (C) total voided volume were measured in a metabolic cage. *P < 0.05, **P < 0.01 and ***P < 0.001 versus vehicle control (One-way ANOVA test).

**Fig 3:**
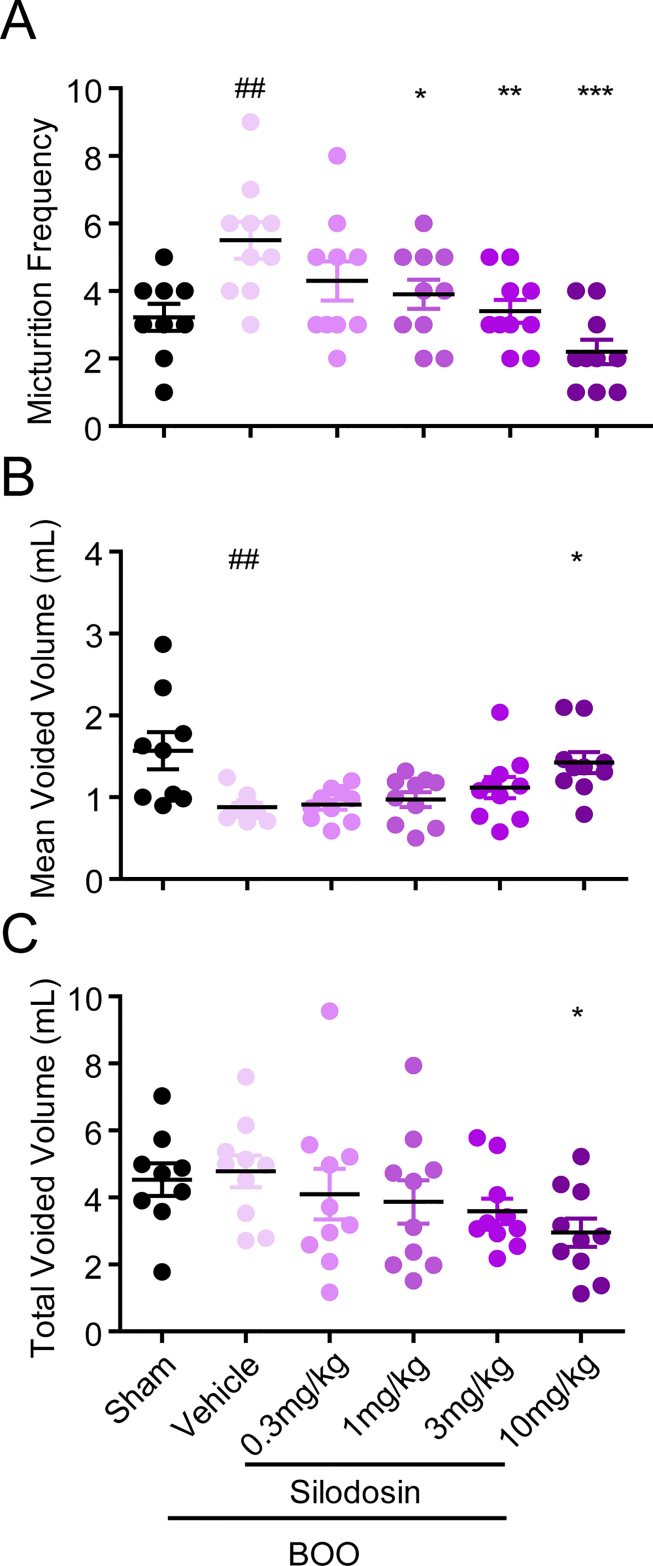
Effects of silodosin on the micturition parameters in BOO rats. BOO rat model was established by partial ligature of the proximal urethra. Voiding behavior was studied after 4 weeks of recovery. Twenty minutes after oral administration of silodosin, distilled water (30 ml/kg) were given orally. (A) Micturition frequency, (B) mean voided volume and (C) total voided volume were measured for the first 2 hours after water load. ^##^P < 0.01 versus sham control (Student t test). *P < 0.05, **P < 0.01 and ***P < 0.001 versus vehicle control (One-way ANOVA test).

The BPH rats were then treated with α_1_-AR antagonist silodosin to evaluate whether this model can be used to test therapeutic drugs aimed to reduce abnormalities in micturition. The results showed that silodosin significantly reduced the urinary frequency and increased the mean voided volume in BPH rats in a dose-dependent manner, with the minimal effective dose of 1 mg/kg (Fig 2A and 2B), while no significant difference was observed in total voided volume between the silodosin-treated and control groups. We also developed the more commonly used model to evaluate urine outlet obstruction, the rat BOO model. Four weeks after the obstruction operation, the mean voiding frequency significantly increased while the mean voided volume significantly decreased in the BOO rats, comparing with the sham-operated rats. Silodosin also significantly reduced the urinary frequency in a dose-dependent manner, with the minimal effective dose of 1 mg/kg (Fig 3A). However, probably due to the surgical obstruction, silodosin was only effective in increasing the mean voided volume at high dose 10 mg/kg, and the total voided volume was also slightly reduced (Fig 3B and 3D) These data indicate that testosterone-induced BPH rat might be a more natural and sensitive model to evaluate drugs targeting the urination obstruction problems than the commonly use BOO rat model.

### Testosterone propionate induces BPH in young male dogs

**Fig 4:**
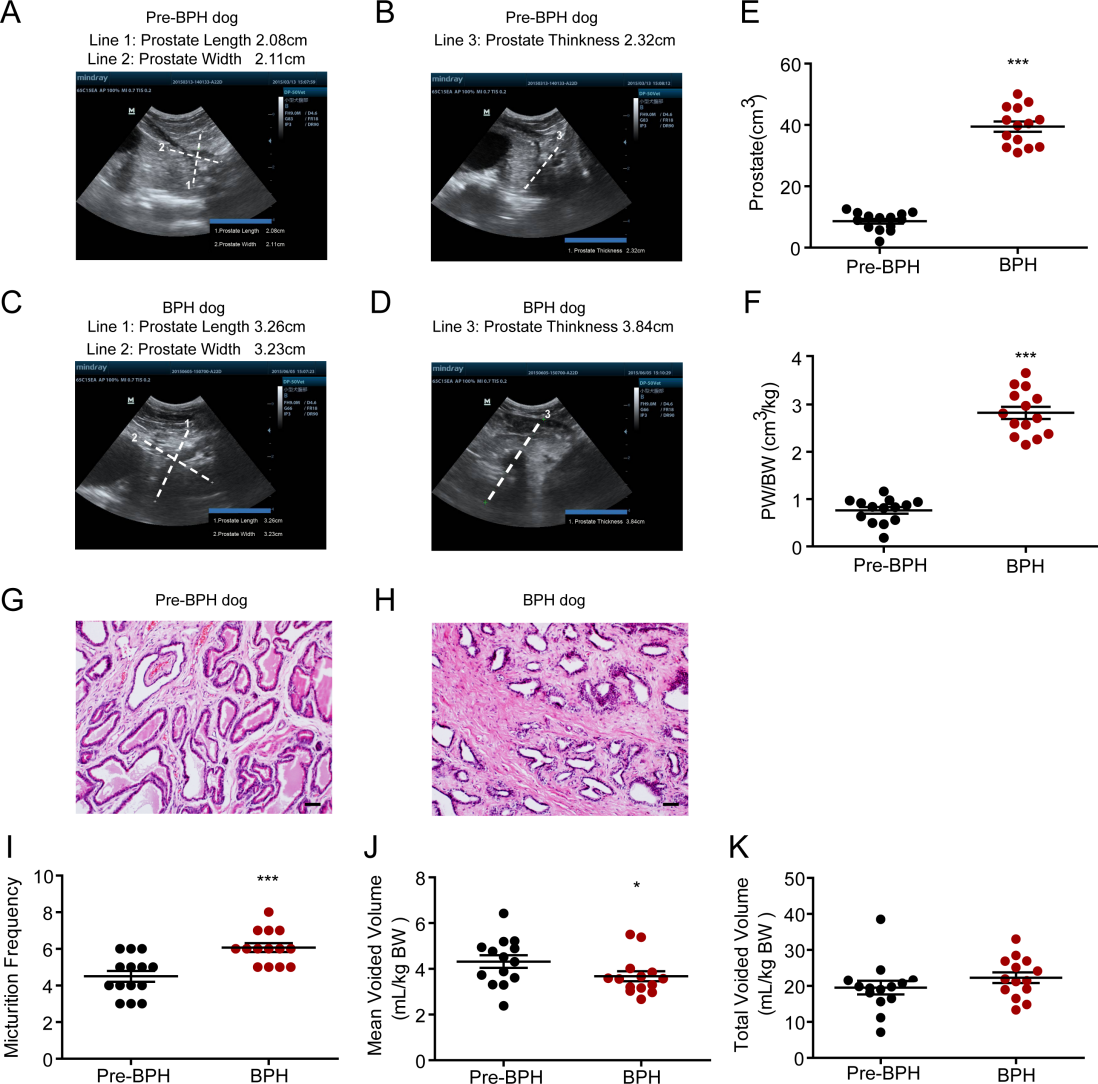
Voiding behavior in testosterone-induced BPH dogs. Prostatic dimensions were obtained with ultrasonic diagnosis instrument before castration (pre-BPH) and 8 weeks after testosterone propionate administration (BPH). (A-D) Representative ultrasound images of the prostates of a pre-BPH dog (A and B) and a testosterone-induced BPH dog (C and D). Line 1, line 2 and line 3 represented the prostate length, width and thickness, respectively. Dog prostate volume = length × width × thickness. The prostate volume (E) and prostate volume to body weight ratio (F) of pre-BPH and testosterone-induced BPH dogs. H&E stains of prostate in pre-BPH and BPH dogs (G and H). (I) Micturition frequency, (J) mean voided volume and (K) total voided volume in the first 2 hours after water load were measured at 8 weeks after the induction of BPH. *P < 0.05 and ***P < 0.001 versus pre-BPH control (Student t test). Scale bar, 50μm.

To establish BPH dog model, young adult male beagle dogs (1 year old, 9–13 kg) were castrated and then subcutaneously injected with testosterone propionate (10 mg/kg per day) for 8 weeks. The prostate volume was measured with ultrasonic diagnosis instrument before castration (pre-BPH group, Fig 4A and 4B) and 8 weeks after testosterone administration (BPH group, Fig 4C and 4D). After basic prostatic dimensions were obtained with ultrasonic diagnosis instrument, the size of prostate gland was calculated from the equation: volume = length× width × thickness. As expected, testosterone propionate treatment significantly increased the prostate volume and prostate volume to body weight ratio (Fig 4E and 4F). As illustrated in Fig 4G and 4H, testosterone treatment characteristically caused hyperplasia in the stroma, which is composed of collagen and smooth muscle, of the dog prostate, but not the epithelia. Micturition behaviors were measured before castration (pre-BPH group) and after the onset of BPH. Similar to testosterone-induced BPH rats, the testosterone-induced BPH dogs have higher micturition frequency (Fig 4G) and lower mean voided volume (Fig 4H) compared to the pre-BPH dogs. There were no significant differences in total voided volume between the BPH and pre-BPH groups (Fig 4I).

### Silodosin relieves voiding symptoms in testosterone-induced BPH dogs

**Fig 5:**
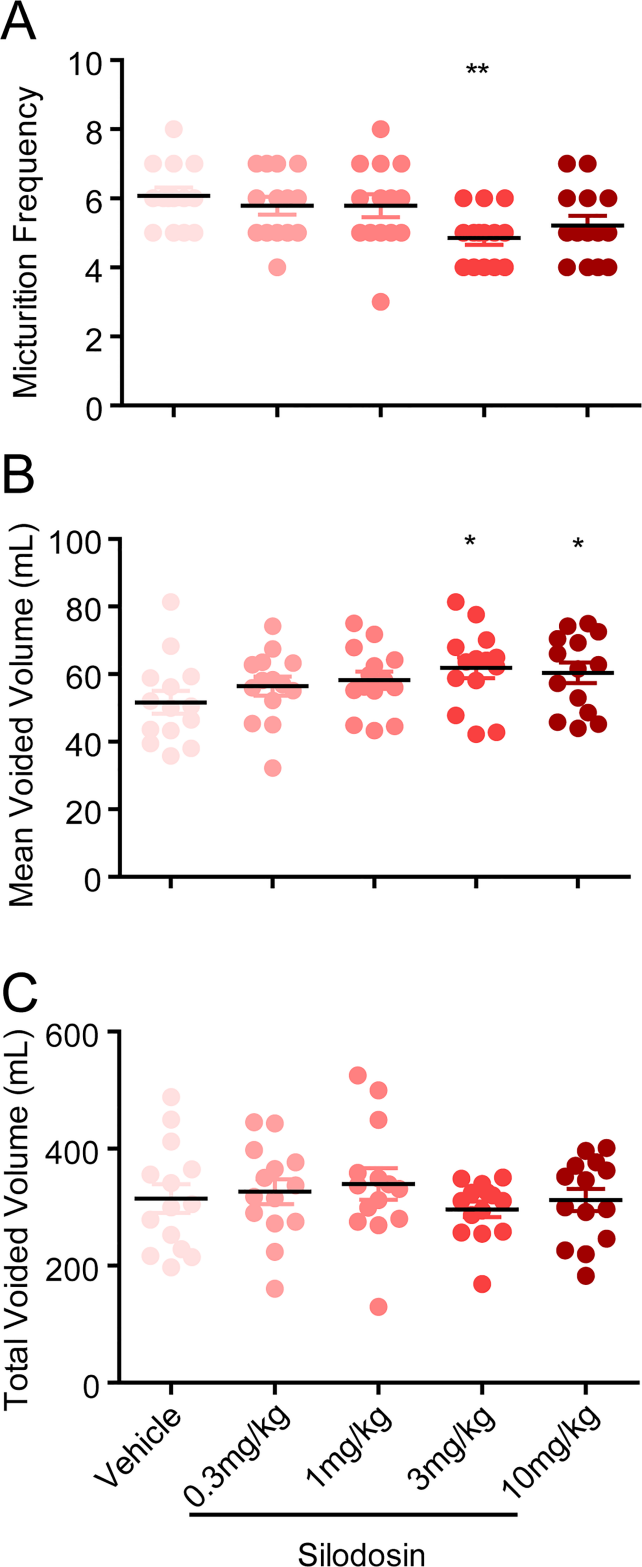
Effects of silodosin on the micturition parameters in testosterone-induced BPH dogs. Twenty minutes after oral administration of silodosin, distilled water (30 ml/kg) were given orally. (A) Micturition frequency, (B) mean voided volume and (C) total voided volume in the first 2 hours after water load were measured in a metabolic cage. *P < 0.05, ***P < 0.001 versus vehicle control (One-way ANOVA test).

The α_1_-AR antagonist silodosin also improved the micturition behavior in testosterone-induced BHP dogs in a dose-dependent manner. The minimal effective dose of reducing micturition frequency and the minimal effective dose of increasing the mean voided volume were both 3 mg/kg (Fig 5A and 5B). There was no significant difference in total voided volume between the silodosin and control groups. These results suggest testosterone-induced BPH dog might be another good model to evaluate drugs targeting the urination obstruction problems.

## Discussion

BPH is a highly prevalent disorder in elderly men, and its incidence rate increases incrementally with age. BPH is associated with voiding and storage LUTS which reduce the life quality of the patients. More serious complications of BPH include acute urinary retention, renal insufficiency, urinary tract infection, gross hemaruria, bladder stones, and renal failure. In the last decade, α_1_-AR has received considerable attention due to the clinical success of α_1_-AR antagonists in the treatment of the symptoms of BPH[21]. In fact, α_1_-AR antagonists are considered one of the first-line therapies to reduce LUTS associated with clinical BPH with proven efficacy [22–24]. The currently marketed α_1_-AR antagonists for the treatment of BPH include terazosin, doxazosin, alfuzosin, tamsulosin, and silodosin[23, 25]. Many published *in vivo* studies of the α_1_-AR antagonists were based on surgically established BOO animal models[20, 25, 26]. BOO is a common problem in BPH patients[13]. Effects similar to BOO in humans are relatively straightforward to replicate surgically in animals. These models display many of the structural and physiological changes in the bladder wall which is also seen in patients, including muscle cell hypertrophy, altered responsiveness to stimuli, increased spontaneous myogenic activity with the development of non-micturition contractions, and enlarged sensory neurons and parasympathetic ganglia[27, 28]. However, in many of the studies concerning the *in vivo* efficacy of α_1_-AR antagonists, female BOO rats were used [20, 23, 25, 26], due to the simplicity of the surgical procedure in female animals. Moreover, the BOO model is an acute model and typically displays severer symptoms than real BPH model.

Therefore, we hereby present hormone-induced BPH rat and dog models, which may be more relevant to the real disease. Since the rat prostate does not surround the urethra, it is believed that rats are unlikely to develop the LUTS seen in the human or dog. However, there is evidence that the overgrowth of the prostate located at the anterior urethra can affect the free passage of urine during micturition[29]. Maggi et al. successfully introduced a rat BPH model which was used to explore the effect of dihydrotestosterone in prostate growth and micturition obstruction in rats[30]. Lee et al. developed a neurohormone-induced BPH rat model to the study the development of obstruction in micturition[29]. Rat prostate can respond to hormone treatment. However, only the dorsal lobe of the rodent prostate is ontogenetically comparable to the human prostate[31]. It has been reported that hormonal treatment not only induces prostate growth but also hardens the ventral lobe of the prostate[32], and such hardening is correlated with the increases in micturition frequency [29]. Our results have shown that the epithelial cell layer and stromal cell space in both the dorsal and ventral lobes of the rat prostate were induced by testosterone. Studies with metabolic cages showed that the voiding behavior of rats with BPH was characterized by an increase in urination frequency and a decrease in voided volume. And α_1_-AR antagonist silodosin effectively alleviated the voiding symptoms of these BPH rats. The testosterone-induced BPH rat seems to be a more sensitive model than the commonly used BOO rat model to evaluate drugs targeting the urination obstruction problems. Silodosin was able to significantly increase voided volume at 1 mg/kg dosage in testosterone-induced BPH rats; however, a much higher dosage (10 mg/kg) must be used to increase voided volume in surgically produced BOO rats. Our findings suggest that both the dorsal and ventral lobes of the rat prostate might contribute to the alteration in voiding behavior in testosterone-induced BPH rats.

We also established a testosterone-induced dog model of BPH and validated its applicability in in vivo efficacy test for α1-AR antagonist. Our results have shown that the prostate stroma increased significantly in testosterone induced BPH dogs. Four histologic patterns of the prostate could be identified in male dogs: immature, normal, benign glandular hyperplasia, and benign complex hyperplasia[33]. In the complex form of BPH in dog, a relative enlargement in the stroma can be observed, which resemble the stromal hyperplasia in BPH human [34–36]. Therefore, our results suggest that there are more histological similarities between the prostate of testosterone-induced BPH dogs and human BPH patients. Silodosin also improved the micturition behavior of these testosterone-induced BHP dogs in a dose-dependent manner. Comparing to the hardly available and very expensive old dogs with spontaneous BPH, these testosterone-induced BHP dogs are very easy to produce and may provide a very sensitive and clinically relevant model for the evaluation of drugs aimed to relief voiding symptoms in BPH. The testosterone’s effect is very robust in dogs, indicates LUTS is worse in BPH dogs than in BPH rats. This may reduce the effectiveness of silodosin in dogs. Considering the bioavailability and PK/PD of silodosin are different in rat, dog and human, it would be difficult to directly compare the dose-effectiveness of a drug cross species. Never the less, our study demonstrated that testosterone-treated dogs could be a useful and more physiological relevant model of BPH, and it could be used to evaluate the effect of new drugs.
